# Evidence for Involvement of Nonclassical Pathways in the Protection From UV‐Induced DNA Damage by Vitamin D–Related Compounds

**DOI:** 10.1002/jbm4.10555

**Published:** 2021-09-29

**Authors:** Warusavithana Gunawardena Manori De Silva, Jeremy Zhuo Ru Han, Chen Yang, Wannit Tongkao‐On, Bianca Yuko McCarthy, Furkan Akif Ince, Andrew J.A. Holland, Robert Charles Tuckey, Andrzej T. Slominski, Myriam Abboud, Katie Marie Dixon, Mark Stephen Rybchyn, Rebecca Sara Mason

**Affiliations:** ^1^ Physiology, School of Medical Sciences and Bosch Institute University of Sydney Sydney NSW Australia; ^2^ Anatomy & Histology, School of Medical Sciences and Bosch Institute University of Sydney Sydney NSW Australia; ^3^ Department of Paediatric Surgery, The Children's Hospital at Westmead University of Sydney Sydney NSW Australia; ^4^ School of Molecular Sciences The University of Western Australia Perth WA Australia; ^5^ Department of Dermatology University of Alabama at Birmingham Birmingham AL USA; ^6^ Zayed University Dubai United Arab Emirates; ^7^ School of Chemical Engineering University of NSW Sydney NSW Australia; ^8^ School of Life and Environmental Sciences University of Sydney Sydney NSW Australia

**Keywords:** 1α,25‐DIHYDROXYVITAMIN D_3_, LUMISTEROL, UV‐INDUCED DNA DAMAGE, VITAMIN D RECEPTOR, ERp57, CREB PHOSPHORYLATION, OXIDATIVE PHOSPHORYLATION

## Abstract

The vitamin D hormone, 1,25dihydroxyvitamin D_3_ (1,25(OH)_2_D_3_), and related compounds derived from vitamin D_3_ or lumisterol as a result of metabolism via the enzyme CYP11A1, have been shown, when applied 24 hours before or immediately after UV irradiation, to protect human skin cells and skin from DNA damage due to UV exposure, by reducing both cyclobutane pyrimidine dimers (CPD) and oxidative damage in the form of 8‐oxo‐7,8‐dihydro‐2′‐deoxyguanosine (8‐OHdG). We now report that knockdown of either the vitamin D receptor or the endoplasmic reticulum protein ERp57 by small, interfering RNA (siRNA) abolished the reductions in UV‐induced DNA damage with 20‐hydroxyvitamin D_3_ or 24‐hydroxylumisterol_3,_ as previously shown for 1,25(OH)_2_D_3_. Treatment with 1,25(OH)_2_D_3_ reduced oxygen consumption rates in UV‐exposed and sham‐exposed human keratinocytes and reduced phosphorylation of cyclic AMP response binding element protein (CREB). Both these actions have been shown to inhibit skin carcinogenesis after chronic UV exposure, consistent with the anticarcinogenic activity of 1,25(OH)_2_D_3_. The requirement for a vitamin D receptor for the photoprotective actions of 1,25(OH)_2_D_3_ and of naturally occurring CYP11A1‐derived vitamin D–related compounds may explain why mice lacking the vitamin D receptor in skin are more susceptible to UV‐induced skin cancers, whereas mice lacking the 1α‐hydroxylase and thus unable to make 1,25(OH)_2_D_3_ are not more susceptible. © 2021 The Authors. *JBMR Plus* published by Wiley Periodicals LLC on behalf of American Society for Bone and Mineral Research.

## Introduction

Vitamin D_3_ is primarily made in skin through the absorption of UVB photons by 7‐dehydrocholesterol, opening the B‐ring of the sterol to form pre‐vitamin D_3_. At body temperature, pre‐vitamin D3 isomerizes to vitamin D3.^(^ ^1^ ^)^ However, the same sunlight exposure that produces vitamin D_3_ also causes several types of DNA damage in skin cells.^(^ ^2^, ^3^ ^)^ Some DNA bases directly absorb photons. Upon UV absorbance, adjacent pyrimidine bases of the same DNA strand form dimeric photolesions.^(^ ^4^, ^5^ ^)^ The most prevalent UV‐induced lesions are the cis‐syn cyclobutane pyrimidine dimers (CPDs), mostly formed between the 5–6 bonds of adjacent thymine and cytosine pyrimidines.^(^ ^6^ ^)^ The commonest forms of CPD are thymine dimers, which are present in numbers proportional to total CPD of all types.^(^ ^7^ ^)^ Although thymine dimers (even if not properly repaired) are not in theory mutagenic, they have been shown to cause mutations in practice.^(^ ^8^, ^9^ ^)^ Thymine‐cytosine dimers and cytosine‐cytosine dimers are highly mutagenic if not correctly repaired by nucleotide excision repair.^(^ ^9^ ^)^

Indirect DNA damage or oxidative damage to purine bases has been shown to contribute to mutagenesis, cancer, aging, and other pathological conditions.^(^ ^10^ ^)^ The main endogenous agents that cause this damage are free radicals such as reactive oxygen species (ROS)^(^ ^11^ ^)^ and reactive nitrogen species (RNS).^(^ ^12^ ^)^ Oxidative DNA damage, with free radicals targeting guanine, produces the main photolesion, 8‐oxo‐7,8‐dihydro‐2′‐deoxyguanosine (8‐oxoguanine/8‐OHdG).^(^ ^13^ ^)^ 8‐OHdG is used as a biomarker for DNA damage by oxidative stress. Oxidative stress may cause noncanonical base pairing and incorrect pairing by DNA polymerase, leading to DNA damage,^(^ ^8^, ^9^ ^)^ mutations,^(^ ^9^, ^14^ ^)^ and contributing to photocarcinogenesis.^(^ ^15^ ^)^

**Fig. 1:**
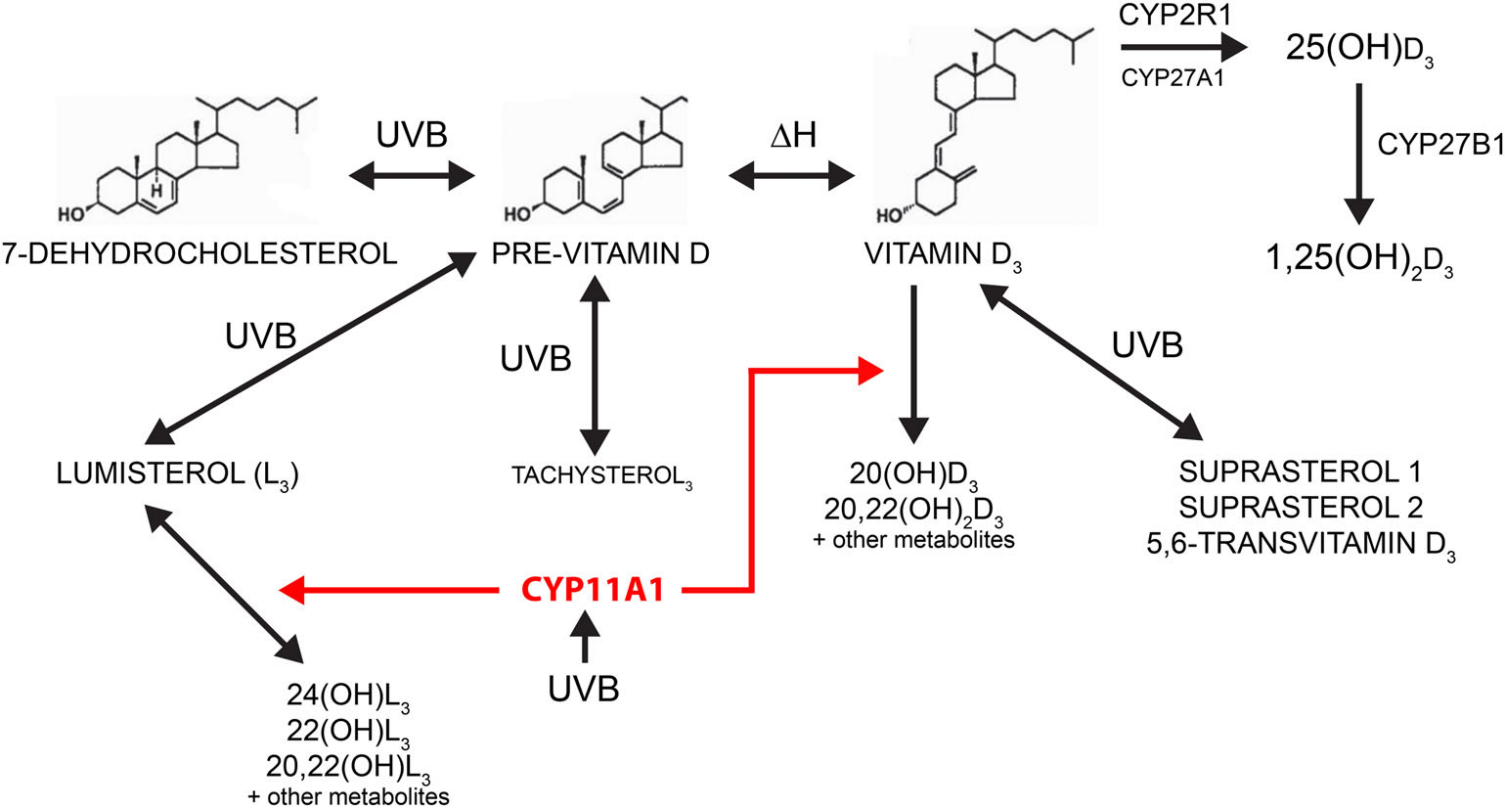
Production and metabolism of vitamin D_3_ and related compounds in the skin. Pre‐vitamin D_3_ is synthesized when the B‐ring of 7‐dehydrocholesterol is broken on the absorption of a photon of UVB. At body temperature, pre‐vitamin D3 is converted to vitamin D_3_. Continued absorption of UV photons by pre‐vitamin D or vitamin D_3_ results in conversion to overirradiation products such as lumisterol_3_, tachysterol_3_, or suprasterols, or 5,6‐transvitamin D_3_.Vitamin D_3_ is converted in the skin to 25‐hydroxyvitamin D_3_ by CYP2R1 (or possibly CYP27A1) and then to 1,25‐dihydroxyvitamin D_3_ by CYP27B1. The cholesterol side‐chain cleavage enzyme CYP11A1 is also expressed in the skin and upregulated by UV. It can convert vitamin D_3_ into 20‐hydroxyvitamin D_3_ and at least 10 other products. CYP11A1 can also convert lumisterol_3_ into 24‐hydroxylumisterol_3_ and several other lumisterol derivatives. (Adapted from Tuckey^(^ ^25^ ^)^).

Pre‐vitamin D_3_ and vitamin D_3_ are not the only vitamin D compounds made in skin. Vitamin D_3_ is metabolized in skin cells through 25‐hydroxyvitamin D_3_ (25(OH)D_3_) to the active hormone, 1,25‐dihydroxyvitamin D_3_ (1,25(OH)_2_D_3_).^(^ ^16^, ^17^ ^)^ Vitamin D_3_ in skin can also be metabolized by the enzyme CYP11A1,^(^ ^18^, ^19^ ^)^ which increases in expression after exposure to UV,^(^ ^20^ ^)^ to produce 20‐hydroxyvitamin D_3_ (20(OH)D_3_) and other compounds (Fig. 1).^(^ ^21^, ^22^, ^23^ ^)^ Furthermore, continued absorption of UV by pre‐vitamin D_3_ produces so called, “overirradiation products,” including the major product, lumisterol.^(^ ^24^ ^)^ Lumisterol can also be metabolized by CYP11A1 to produce several hydroxylated metabolites including 24‐hydroxylumisterol_3_ (24(OH)L_3_) (Fig. 1).^(^ ^25^, ^26^ ^)^

Sunlight produces both vitamin D3 and DNA damage. Although DNA damage occurs relatively quickly, from immediately to a few hours,^(^ ^27^, ^28^ ^)^ production of vitamin D3 takes many hours.^(^ ^1^ ^)^ This gives a window of opportunity to test whether vitamin D compounds added topically, either before UV exposure, or in most cases immediately after UV exposure, could reduce UV‐induced DNA damage. Many studies, including studies carried out with Tony Norman (deceased; Anthony W. Norman, PhD, Department of Biochemistry, University of California, Riverside, Riverside, California, USA) reported that the vitamin D hormone, 1,25(OH)_2_D_3_, reduced UV‐induced DNA damage in skin cells, skin explants and in human subjects.^(^ ^28^, ^29^, ^30^, ^31^, ^32^, ^33^, ^34^, ^35^, ^36^, ^37^, ^38^ ^)^ Vitamin‐D like compounds such as 20(OH)D_3_ and the synthetic compound 1,25‐dihydroxylumisterol_3_, synthesized by Bill Okamura in Tony Norman's group,^(^ ^39^ ^)^ also have been shown to reduce UV‐induced CPD in separate studies.^(^ ^33^, ^34^, ^38^, ^40^, ^41^, ^42^, ^43^ ^)^ It seems likely then, that vitamin D compounds, synthesized in skin as a result of sunlight exposure, contribute to protection of DNA in skin cells from the next exposure to UV. This skin adaptation is similar in timing to the development of a thickened stratum corneum,^(^ ^44^ ^)^ the outer layer of skin that attenuates UV penetration,^(^ ^45^ ^)^ and pigmentation (tan), both of which contribute to reduced UV damage and photocarcinogenesis.^(^ ^46^, ^47^ ^)^

Indeed, in mice, topical application of 1,25(OH)_2_D_3_ immediately after UV exposure over 10 weeks, significantly reduced skin tumor development over the subsequent 30 weeks.^(^ ^34^ ^)^ 1,25(OH)_2_D_3_ normally requires the vitamin D receptor (VDR), a member of the steroid hormone receptor superfamily, to effect changes in cellular function.^(^ ^48^, ^49^ ^)^ In support of the hypothesis that 1,25(OH)_2_D_3_ contributes to skin adaptation to sunlight exposure, mice with a nonfunctional VDR developed more skin tumors than wild‐type (WT) mice after UV exposure^(^ ^50^ ^)^ or after oral administration of a chemical carcinogen.^(^ ^51^ ^)^ Somewhat surprisingly, mice with ablation of CYP27B1, the 1α‐hydroxylase that converts 25‐hydroxyvitamin D to 1,25(OH)_2_D_3_, are not more susceptible to UV‐induced skin tumors.^(^ ^52^ ^)^ This raises the likelihood that other vitamin D compounds made in skin (Fig. 1) may also contribute to protection of DNA from UV exposure. There is some evidence to support this proposal,^(^ ^38^, ^41^, ^42^, ^43^, ^53^ ^)^ but more is needed.

1,25(OH)_2_D_3_ binds to the VDR, which acts principally as a modifier of transcription in the nucleus.^(^ ^54^ ^)^ Tony Norman's group was the first to demonstrate a nonclassical pathway of 1,25(OH)_2_D_3_ response: a rapid intestinal calcium uptake response to 1,25(OH)_2_D_3_ was shown to be via plasma membrane‐associated VDR and/or endoplasmic reticulum protein, ERp57, a member of the protein disulphide isomerase family (PDIA3).^(^ ^55^, ^56^, ^57^, ^58^, ^59^ ^)^ The receptor has been called 1,25D3–membrane associated rapid response steroid binding (1,25D3‐MARRS).^(^ ^60^, ^61^ ^)^ This nonclassical pathway was also shown to be present in bone cells.^(^ ^59^, ^62^, ^63^ ^)^

In collaboration with Tony Norman, we published evidence that, at least in part, the protective effect of 1,25(OH)_2_D_3_ to reduce UV‐induced CPD depends on both VDR and ERp57.^(^ ^64^ ^)^ These earlier studies in skin cells from patients with Hereditary Vitamin D Resistant Rickets type 1, due to anomalies in the VDR,^(^ ^65^, ^66^, ^67^ ^)^ showed that 1,25(OH)_2_D_3_ did not reduce CPD in fibroblasts from a patient with an early stop codon in the *VDR* gene, but was effective in reducing CPD in fibroblasts from patients with mutations in either the DNA binding domain or the ligand binding domain.^(^ ^64^ ^)^ Treatment of skin fibroblasts with a neutralizing antibody (which would not be expected to pass across the cell membrane) to ERp57 or small, interfering RNA (siRNA) to ERp57 also abolished the protective effect of 1,25(OH)_2_D_3_ on CPD after UV.^(^ ^64^ ^)^ Immunoprecipitation experiments on non‐nuclear fractions of these skin cells revealed a VDR‐ERp57 complex. Furthermore, the use of 4,40‐diisothiocyanatostilbene‐2,20‐disulfonic acid (DIDS), a chloride channel blocker shown to prevent 1,25(OH)_2_D_3_–induced chloride currents in osteoblasts,^(^ ^68^ ^)^ also blocked the reduction in post‐UV CPD by 1,25(OH)_2_D_3._^(^ ^37^ ^)^

But there are many unknowns. Although 20(OH)D_3_ and its metabolites derived from vitamin D3 via CYP11A1 reduce both CPD and oxidative DNA damage in the form of 8‐OHdG,^(^ ^38^, ^42^, ^69^ ^)^ there is a shortage of similar information on the CYP11A1 derivatives of pre‐vitamin D, such as 24(OH)L_3_. Recent studies, however, have demonstrated a photoprotective role for CYP11A1‐derived hydroxylumisterol compounds in human keratinocytes.^(^ ^69^, ^70^ ^)^ In addition, although a nonfunctional VDR or knockdown of ERp57 reduced UV‐induced CPD in skin fibroblasts, it is not known whether knockdown of VDR or ERp57 abolishes the protection against UV‐induced CPD by 1,25(OH)_2_D_3_ in keratinocytes, the main epidermal cell type. Nor is there data on whether VDR or ERp57 are also required for protection by 1,25(OH)_2_D_3_ against oxidative DNA damage or whether any protection against DNA damage by CYP11A1‐derived metabolites of vitamin D3, like 20(OH)D_3_, or of pre‐vitamin D3, like 24(OH)L_3_, requires VDR and/or ERp57. Furthermore, evidence has accumulated for vitamin D hydroxyderivatives acting on alternative nuclear receptors including retinoic acid orphan receptor (ROR)α and γ,^(^ ^71^ ^)^ aryl hydrocarbon receptor (AhR),^(^ ^72^ ^)^ and liver X receptors (LXR).^(^ ^73^ ^)^ These questions and the possibility that 1,25(OH)_2_D_3_ affects some keratinocyte functions known to play a role in photocarcinogenesis were tested in the current study.

## Subjects and Methods

### Vitamin D compounds

24(OH)L_3_ and 20(OH)D_3_ were enzymatically synthesized using recombinant CYP11A1, procedures as described.^(^ ^21^, ^26^ ^)^ Other compounds were purchased: 1,25(OH)_2_D_3_ (Sapphire Bioscience, Sydney, Australia). All compounds were dissolved in spectroscopic grade ethanol (Merck, Darmstadt, Germany) and stored under argon gas at −80°C. The absorbance of these compounds was determined by Nanodrop ND‐1000 Spectrophotometer (Thermo Fisher Scientific, Waltham, MA, USA), and concentration was calculated using the Beer‐Lambert law. The final concentration of ethanol vehicle used in experiments was 0.1% vol/vol.

### Solar simulated irradiation

Cells were exposed to solar simulated UV radiation (ssUV), provided by a Oriel Sol1A™ 94042A 450W solar simulator (Newport Corporation, Irvine, CA, USA) with an inbuilt attenuation filter to eliminate UVC. UVA and UVB output was calibrated using a OL756 spectroradiometer (Optronic Laboratories, Gooch & Housego, Orlando, FL, USA) as described.^(^ ^37^ ^)^ The UV dose was determined from previous studies to cause reasonable levels of DNA damage in human keratinocytes, but was not high enough to result in a large increases in apoptosis.^(^ ^36^, ^74^ ^)^ Irradiation was carried out as described.^(^ ^37^ ^)^ All treatments with vitamin D compounds were added immediately after exposure to UV.

### Keratinocyte culture

Legal parents or guardians gave written informed consent for collection of skin from circumcision under a protocol approved by the University of Sydney Human Ethics Committee (Reference number 2015/063). Keratinocytes, (passages 1–5) from at least two independent donors were used in all experiments. Keratinocytes were cultured from skin samples and used for studies as described.^(^ ^37^ ^)^ Keratinocytes at passage 2–5 were used for experiments. All experimental work was carried out in Sydney, NSW, Australia.

### siRNA transfection

Opti‐MEM™ Reduced Serum Medium powder (22600134) was from Thermo Fisher Scientific; siRNA transfection reagent, nondirected siRNA sequence (noncomplementary control RNA [siCTRL RNA]), siRNA targeted to VDR (siVDR), and siRNA targeted to ERp57 (siERp57) were all from Santa Cruz Biotechnologies (Dallas, TX, USA). Cell culture media was replaced with 100 μL Opti‐MEM™ serum‐free media (Thermo Fisher Scientific) and incubated at 37°C for 5 to 10 minutes. For VDR, ERp57 knockdown, cells were transfected with 50mM siVDR, siERp57, or siCTRL RNA diluted in siRNA transfection medium/Opti‐MEM™ serum‐free media (Santa Cruz Biotechnologies) in the presence of the recommended concentration of lipid‐based siRNA transfection reagent (Santa Cruz Biotechnologies) according to the manufacturer's instructions, and as described.^(^ ^64^, ^75^ ^)^

### Western blots

Western blots used protein lysates from keratinocytes seeded in six‐well plates at a density of 500,000 cells/well as described.^(^ ^76^ ^)^ Additional antibodies used for protein detection were anti‐ERp57 (mouse monoclonal; Santa Cruz Biotechnologies), anti‐VDR (mouse monoclonal; Santa Cruz Biotechnologies), anti‐phospho–cyclic AMP response binding element protein (CREB)‐Ser^133^ (mouse monoclonal; Cell Signaling Technology, Danvers, MA, USA), and anti‐tubulin at 1 μg/mL (mouse monoclonal; Santa Cruz Biotechnologies).

### Immunochemistry

Cells were incubated for 3 hours at 37°C, based on previous studies,^(^ ^34^, ^37^ ^)^ followed by fixation with ice‐cold 100% methanol at −20°C for 5 minutes and washed three times with MiliQ water (Mili‐Q integral water purification system®; Millipore SAS, Molsheim, France). Cells were left to air‐dry overnight before staining for DNA damage in the form of thymine dimers as an index of CPDs^(^ ^7^ ^)^ or 8‐OHdG, as described.^(^ ^27^ ^)^ Briefly, cells were first treated with 1% H_2_O_2_ (vol/vol in PBS) for 5 minutes to block endogenous peroxidase activity, while covered in foil, followed by three washes with MiliQ water. Antigen retrieval involved nuclear DNA denaturation with 70mM NaOH diluted in 70% ethanol, followed by aspiration and proteolytic digestion with Proteinase K (Final concentration: 1 μg/mL in 0.1mM CaCl_2_) at room temperature for 5 minutes for CPD or 10 minutes at 37°C for 8‐OHdG. The wells were gently washed with MiliQ water twice. Nonspecific staining was blocked with 50% horse serum (vol/vol in PBS) (Sigma‐Aldrich, St. Louis, MO, USA). Immunohistochemistry was performed using the anti‐thymine dimer antibody (mouse monoclonal clone H3; Sigma‐Aldrich) at 5 μg/mL or the 8‐OHdG antibody (mouse monoclonal lgG2b, clone 15A3; Santa Cruz Biotechnologies) at 2 μg/mL overnight at 4°C, followed by incubation at room temperature with goat anti‐mouse immunoglobulin G (IgG) F(ab′)2 secondary antibody, biotin conjugate at 1:500 (Thermo Fisher Scientific) for 20 minutes at room temperature, and horseradish peroxidase (HRP)‐Streptavidin conjugate at 1:150 (Invitrogen, Carlsbad, CA, USA) for 15 minutes at room temperature. Isotype control was performed with mouse IgG instead of primary antibody. All steps were separated by washing steps with Tween‐PBS (PBST) for three times. HRP substrate diaminobenzidine (DAB) (Enhanced Liquid substrate System for Immunohistochemistry; Sigma‐Aldrich) was applied for 5 minutes to visualize the staining of DNA damage. Coverslips were rinsed with MiliQ water and mounted with Entellin (Merck) onto glass slides after being air‐dried.

Mouse monoclonal IgG1 ERp57 antibody and mouse monoclonal IgG2a VDR antibody (both from Santa Cruz Biotechnologies) were used for immunohistochemistry of the VDR and ERp57. Cells were fixed with 10% (vol/vol) formaldehyde in PBS for 5 minutes at room temperature and permeabilized with 0.1% (vol/vol) Triton‐X in PBS for 3 minutes at room temperature. Endogenous peroxidase activity was blocked by 6% (vol/vol) H_2_O_2_ in PBS for 10 minutes. Nonspecific antibody binding was inhibited by 10% (vol/vol) horse serum (blocker) in PBS for 1 hour. VDR and ERp57 antibodies in 10% horse serum at 2.5 μg/mL concentration were incubated with the cells overnight at 4°C in blocker. Isotype control antibodies were used under similar experimental concentrations and conditions. Cells were rinsed with PBS five times and incubated with anti‐mouse IgG HRP‐linked antibody in blocker for 1 hour at room temperature. Cells were rinsed with PBS, incubated with DAB and rinsed with Mili‐Q water and mounted as described in the previous paragraph for analysis.

Brightfield images were taken on the Stereo Investigator Scope (MBF Bioscience, Williston, VT, USA) or Zeiss‐Axioscan light microscope (Zeiss, Oberkochen, Germany) at the Bosch Advanced Microscopy Facility at The University of Sydney. The image was thresholded in ImageJ imaging software (NIH, Bethesda, MD, USA; https://imagej.nih.gov/ij/) against the isotype control, which showed low staining as reported.^(^ ^34^ ^)^ Thus, the threshold was set based on the non‐irradiated sample (SHAM) to pick up minimum mean gray value. Mean gray value is the average gray value within the selected area, calculated as the sum of the gray values of all the pixels divided by the number of pixels. The set threshold was applied to all the images. The average of the mean gray value for each coverslip was obtained from 10 fixed‐area regions, where cells were well attached, as reported.^(^ ^36^ ^)^

These immunohistochemical methods for determining CPD and 8‐OHdG after UV have been validated with COMET assays (otherwise known as single‐cell gel electrophoresis), which use specific endonucleases to detect CPD or 8‐OHdG followed by single cell electrophoresis to detect DNA strand breaks.^(^ ^27^, ^37^ ^)^

### Seahorse energetics

The cellular oxygen consumption rates (OCR) were determined in an XF96 Extracellular Flux Analyzer (Seahorse Bioscience, Agilent, Santa Clara, CA, USA) immediately following irradiation or sham irradiation of cells. 1,25(OH)_2_D_3_ at 10nM (or vehicle) was injected immediately after irradiation (earliest possible); *n* = 22–24 per treatment. Data was analyzed using Wave 2.3 (Seahorse Bioscience) and Prism (GraphPad Software, Inc., La Jolla, CA, USA).^(^ ^36^ ^)^

### ROS detection

ROS levels were measured using the ROS‐Glo™ H2O2 assay (Promega, San Luis Obispo, CA, USA) as described.^(^ ^36^ ^)^

### Tunicamycin studies

Tunicamycin (Sigma‐Aldrich, St. Louis, MO, USA), an N‐linked glycosylation inhibitor, was added into the medium at 1 μg/mL, based on previous optimization experiments, and incubated with cells for 48 hours. This was followed by ssUV irradiation and processing for CPD or 8‐OHdG at 3 hours, as described above in Immunohistochemistry.

### Statistical analysis

Keratinocyte experiments were performed in triplicate or as otherwise indicated and experiments were repeated at least twice with different tissue donors. Data were graphed and analyzed with use of GraphPad Prism software. Unless stated otherwise, results are presented as mean + standard error of the mean (SEM) and significance between treatment groups was analyzed with one‐way ANOVA with Tukey posttest.^(^ ^36^ ^)^

## Results

**Fig. 2:**
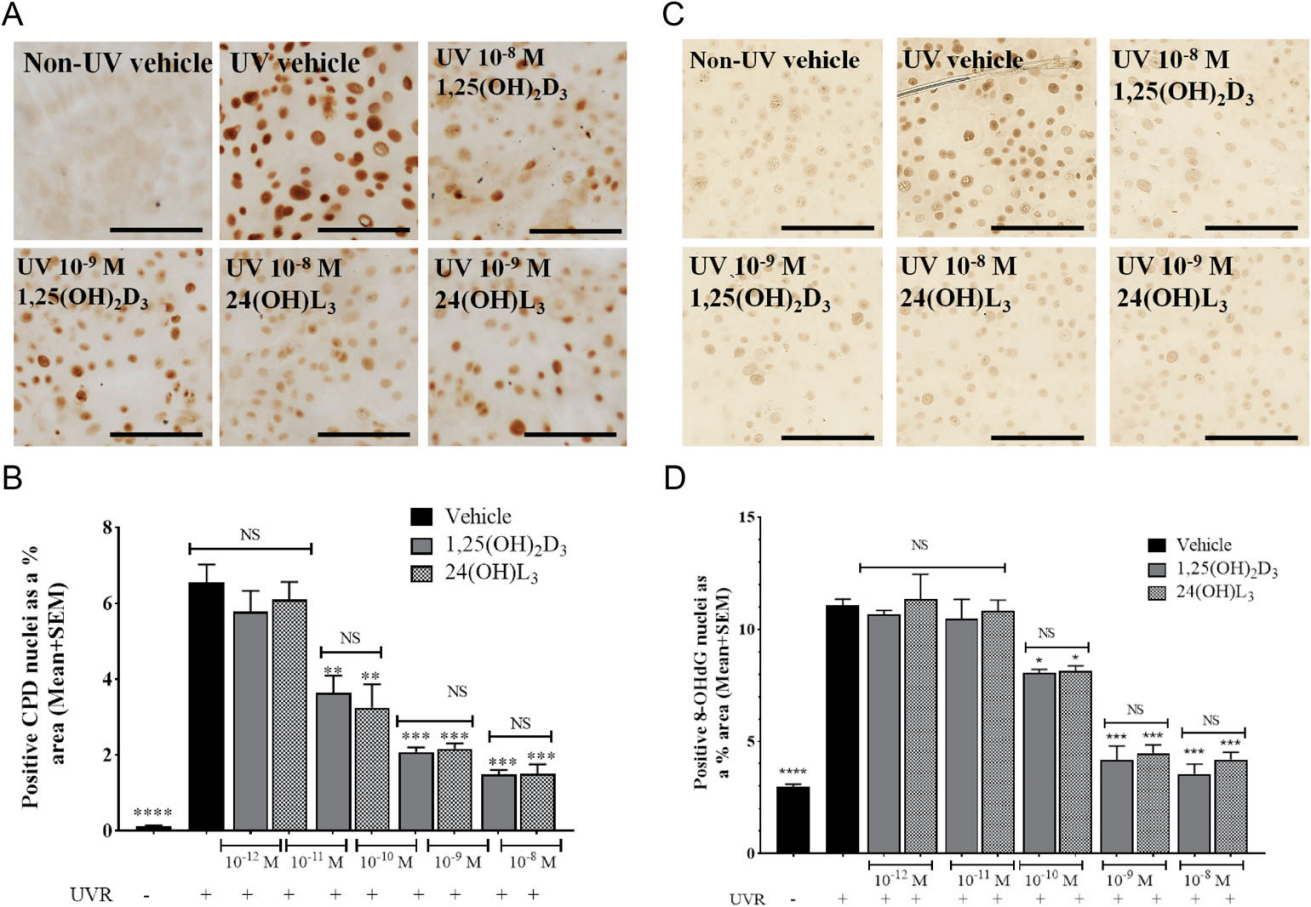
Concentration‐dependent reduction in CPD or 8‐OHdG by 24(OH)L_3_ or 1,25(OH)_2_D_3_ in human primary keratinocytes. Representative photomicrographs of immunohistochemical staining of UV‐induced CPD (*A*) or 8‐OHdG (*C*), 3 hours after treatment with vehicle [0.1% (vol/vol) ethanol], for five concentrations of 1,25(OH)_2_D_3_ or 24(OH)L_3_. Dark brown staining, as shown by arrows, indicates the presence of CPD‐positive nuclei (scale bar = 100 μm). (*B*) Image analysis of IHC images presented as CPD‐positive nuclei (*B*) or 8‐OHdG–positive nuclei (*D*) as a percentage of total area. Solid gray bars show 1,25(OH)_2_D_3_ whereas stippled bars show 24(OH)L_3_. Results are from a single experiment performed in triplicate, representative of three separate experiments with similar results, mean + SEM; **p < 0.01, ***p < 0.001, ****p < 0.0001, when compared to UV vehicle. IHC = immunohistochemistry; NS = not significant between data sets.

Like 1,25(OH)_2_D_3_ and 20(OH)D_3_,^(^ ^38^, ^41^ ^)^ 24(OH)L_3_ reduced UV‐induced CPD and 8‐OHdG in the nuclei of human primary keratinocytes in a concentration‐dependent manner (Fig. 2*A‐D*).

**Fig. 3:**
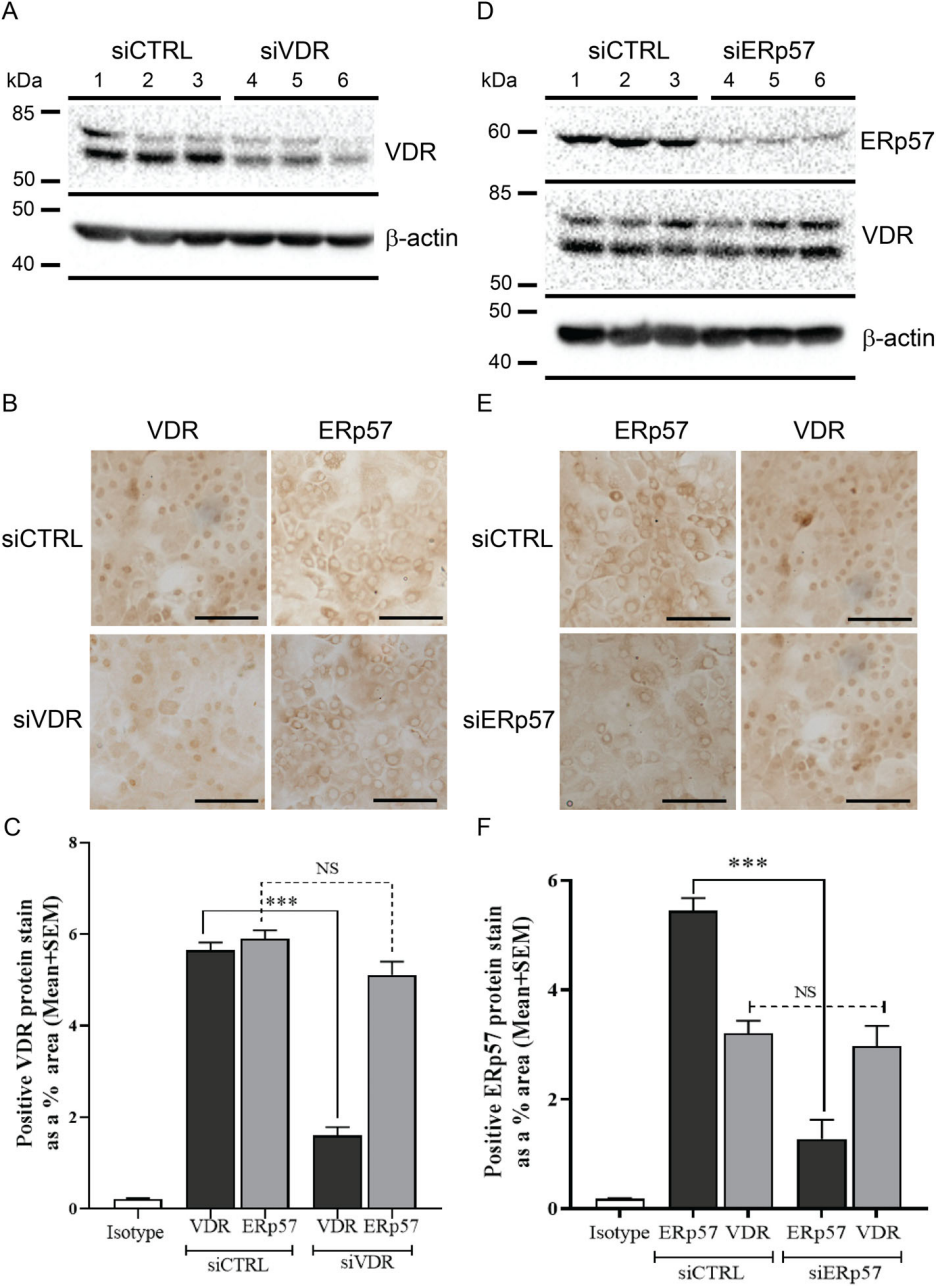
VDR or ERp57 knockdown by siRNA reduced VDR or ERp57 protein expression, respectively, but did not affect the expression of the non‐knocked‐down protein. Representative Western blots showing VDR (*A*) or ERp57 and VDR (*D*) in human primary keratinocytes 48 hours following transfection with siRNA targeted to VDR mRNA (siVDR) (*A*, lanes 4–6), siRNA targeted to ERp57 (siERp57) (*D*, lanes 4–6), or a nondirected siRNA sequence (siCTRL) (*A*, *D*, lanes 1–3). β‐actin is shown as the loading control. Representative photomicrographs of keratinocytes stained for VDR or ERp57 following transfection with siVDR (*B*) or siERp57 (*E*) or siCTRL. Image analysis of IHC images presented as positive VDR (*C*) or ERp57 (*F*) protein expression as a percentage of total cellular area 48 hours after transfection with siVDR, siERp57, or siCTRL. Results were from a single experiment performed in triplicate, representative of two separate experiments with similar results. Mean + SEM; ***p < 0.001, when compared to siCTRL vehicle. IHC = immunohistochemistry; NS = not significant between data sets.

To test whether the VDR or ERp57 was required for the reduction in CPD or oxidative DNA damage by 1,25(OH)_2_D_3_ or 24(OH)L_3_, cells were transfected with siRNA targeted to VDR mRNA (siVDR) or with siRNA to ERp57 (siERp57) or with a nondirected siRNA sequence (siCTRL). VDR or ERp57 knockdown by siRNA was verified by Western blot (Fig. 3*A* for VDR, or Fig. 3*D* for ERp57) and, as an additional control, by immunohistochemistry and image analysis for VDR or ERp57 (Fig. 3*B*, *C, E, F*). Significant reductions in VDR protein expression were observed in cells transfected with siVDR compared to siCTRL (***p < 0.001) (Fig. 3*B*, *C*), similar to Western blot results (Fig. 3*A*), but there were no significant differences observed in ERp57 expression between either siVDR or siCTRL samples. Likewise, significant reductions in ERp57 protein expression were observed in cells transfected with siERp57 compared to siCTRL (***p < 0.001) (Fig. 3*E, F*), similar to Western blot results (Fig. 3*D*), but there were no significant differences observed in VDR expression between either siERp57 or siCTRL samples.

**Fig. 4:**
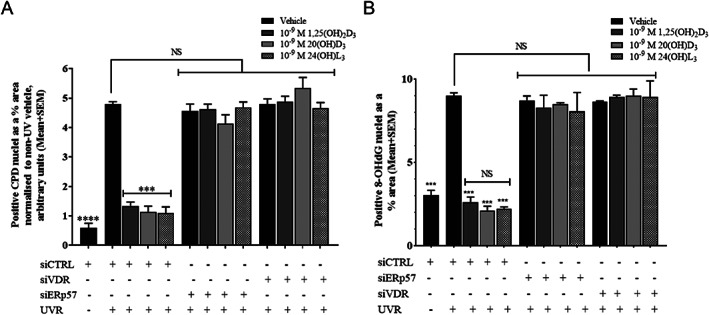
Reductions in UV‐induced CPD or 8‐OHdG by 1,25(OH)_2_D_3_, 20(OH)D_3_, or 24(OH)L_3_ were abolished by either VDR or ERp57 knockdown in human primary keratinocytes. Image analysis of IHC images of CPD (*A*) or 8‐OHdG (*B*). Results are each from a single experiment performed in triplicate, representative of three separate experiments with similar results. Data are presented as positive nuclei as a percentage of total area. Mean + SEM; ****p < 0.0001, ***p < 0.001, when compared to UV vehicle. IHC = immunohistochemistry; NS = not significant between data sets.

Keratinocytes were exposed to the solar simulator 48 hours after siRNA transfection and immediately treated with 1,25(OH)_2_D_3_, 20(OH)D_3_, or 24(OH)L_3_ at 1 × 10^−9^M. After 3 hours, significant reductions in UV‐induced CPD (Fig. 4*A*) or 8‐OHdG (Fig. 4*B*) resulted from these treatments in cells treated with siCTRL (***p < 0.001). Reductions of either UV‐induced CPD or 8‐OHdG with any of these compounds were abolished in the presence of siVDR or siERp57 (Fig. 4*A*,*B*).

**Fig. 5:**
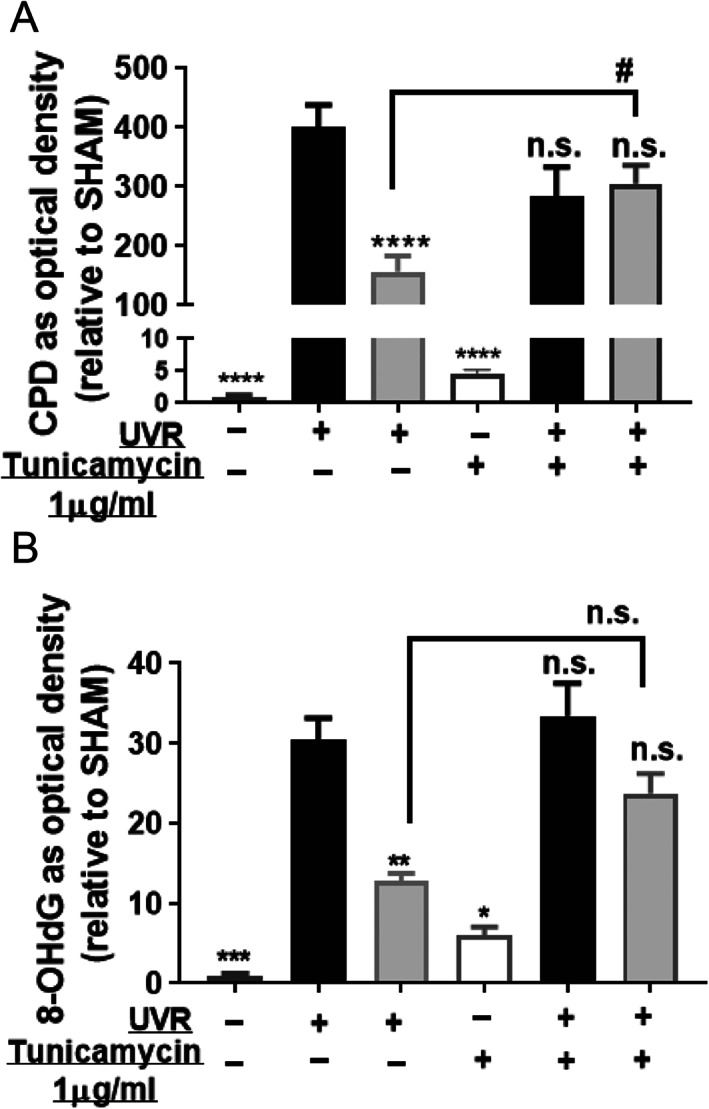
Tunicamycin pretreatment abolished the protective effect of 1,25(OH)_2_D_3_ on UV‐induced CPD and 8‐OHdG. Keratinocytes were treated with 1 μg/mL tunicamycin or vehicle (DMSO) for 48 hours followed by exposure to UV and treatment with vehicle or 1,25(OH)_2_D_3_ (1 × 10^−9^M). (*A*) CPD or (*B*) 8‐OHdG (y‐axis) are shown as optical density relative to SHAM. Open white bars show vehicle without UVR; solid black bars show vehicle with UVR; light gray bars show 1,25(OH)_2_D_3_ with UVR. Values are means + SEM. Results are from three independent experiments each in triplicate (*n* = 9). ****p < 0.0001, **p < 0.01, *p < 0.05, and n.s. (not significant compared with UV + Vehicle [non‐tunicamycin treated]) by mixed model analysis and Sidak's multiple comparison posttest. DMSO = dimethylsulfoxide; UVR = ultraviolet radiation.

In a separate set of experiments, keratinocytes were treated with tunicamycin, an N‐glycosylation inhibitor,^(^ ^77^ ^)^ at 1 μg/mL for 48 hours. The aim of those experiments was to abolish cell surface expression of the Calcium Sensing Receptor (CaSR),^(^ ^78^ ^)^ which was achieved, in order to test modulators of CaSR. In the experiments, the vitamin D hormone, 1,25(OH)_2_D_3_, was used as a positive control for photoprotection. Rather surprisingly, treatment of keratinocytes with tunicamycin abolished the 1,25(OH)_2_D_3_–induced reductions in UV‐induced CPD and 8‐OHdG (Fig. 5*A,B*).

**Fig. 6:**
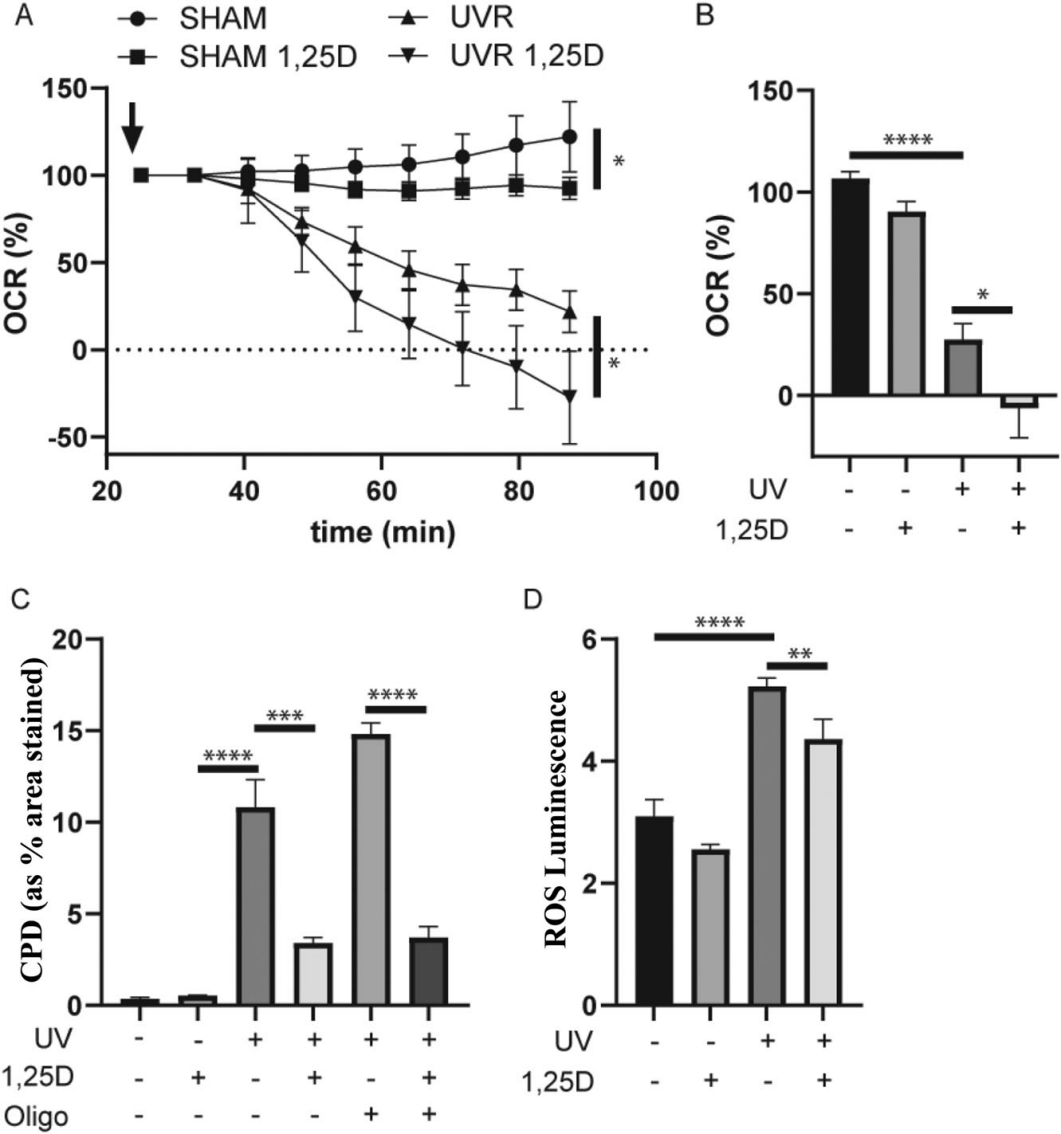
1,25(OH)_2_D_3_ reduced oxygen consumption rate and ROS production under basal conditions and after UV exposure but oxidative phosphorylation was not needed for protection from DNA damage after UV. Cells subjected to Seahorse XF after UV irradiation (“UVR”) or non‐irradiation (“SHAM”) ± 1,25(OH)_2_D_3_ (“1,25D”) (mean ± SEM, *n* = 22). (*A*) OCR following 1,25D/vehicle at injection point (arrow). (*B*) Graph showing OCR percentage 90 minutes after injection. Bars indicate significant differences between groups: ****p < 0.0001, *p < 0.05. (*C*) Graph of CPD in keratinocytes measured by image analysis showing the effect of oligomycin treatment (*n* = 9). (*D*) ROS in arbitrary luminescence units ± UV and or 1,25D (*n* = 9); Bars indicate significant differences between groups: *p < 0.05, **p < 0.01, ***p < 0.001, ****p < 0.0001. CPD = cyclobutane pyrimidine dimers; OCR = oxygen consumption rate; ROS = reactive oxygen species.

UV exposure causes mitochondrial damage,^(^ ^36^, ^79^ ^)^ which reduces oxygen consumption rates as measured by Seahorse analysis (Fig. 6*A*,*B*). In contrast, compounds that reduce oxygen consumption rates without causing mitochondrial damage have been characterized as anti‐carcinogenic.^(^ ^80^ ^)^ Oxygen consumption rates (OCRs), a measure of mitochondrial oxidative phosphorylation, were lower in keratinocytes exposed to 1,25(OH)_2_D_3_ compared with vehicle (Fig. 6*A*). At 90 minutes after UV, OCRs were significantly lower in keratinocytes treated with 1,25(OH)_2_D_3_ (from time shown in arrow), whether SHAM irradiated or exposed to UV (Fig. 6*A*,*B*). Of interest is the observation that treatment with oligomycin, which inhibits oxidative phosphorylation, did not affect the reduction in CPD (Fig. 6*C*) or 8‐OHdG (not shown) caused by the addition of 1,25(OH)_2_D_3_. Production of ROS was significantly increased by UV exposure and significantly reduced in either SHAM or UV‐exposed keratinocytes, in the presence of 1,25(OH)_2_D_3_ (Fig. 6*D*).

**Fig. 7:**
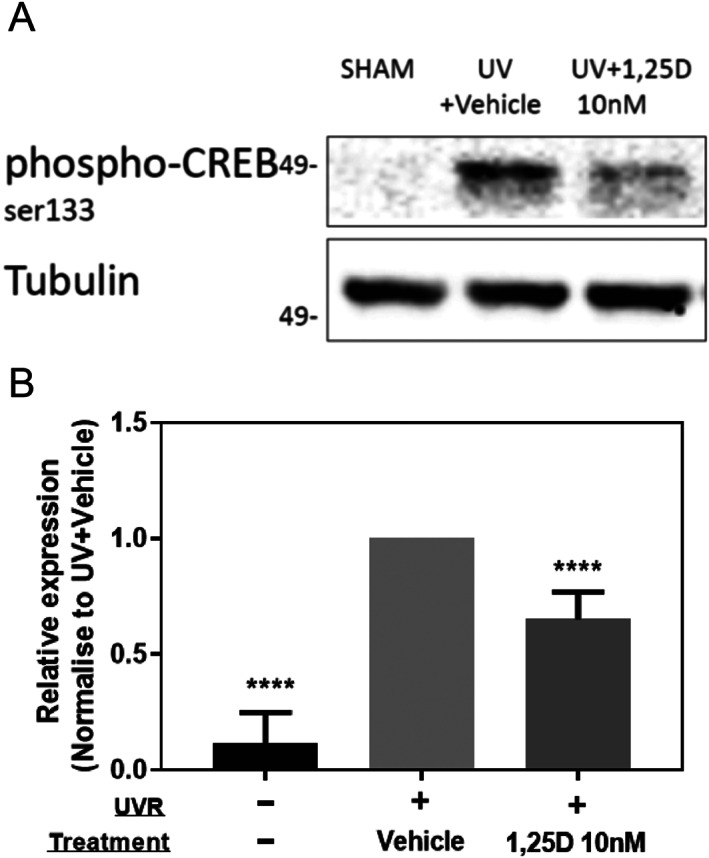
1,25(OH)_2_D_3_ reduced UV upregulated p‐CREB. Human keratinocytes cultured in 96‐well plates were irradiated with 400 mJ/cm^2^ UVB followed by treatment with vehicle or 10nM 1,25(OH)_2_D_3_ for 90 minutes. (*A*) Blot was incubated with anti‐p‐CREB antibody and tubulin, which is shown as a loading control. (*B*) Densitometry of triplicate blots for p‐CREB expression (mean + SD) was normalized to UV + Vehicle and shown as relative expression. ****p < 0.001 when compared with vehicle‐treated cells after UV. p‐CREB = phospho‐CREB.

Increased phosphorylation of CREB is a recently identified marker of (photo)carcinogenic agents.^(^ ^81^ ^)^ As shown in Fig. 7*A,B*, CREB phosphorylation is increased by UV exposure in human keratinocytes, but decreased in UV‐exposed keratinocytes by 1,25(OH)_2_D_3_.

## Discussion

Several studies reported the photoprotective effect of 1,25(OH)_2_D_3_ in reducing UV‐induced DNA damage from *in vitro*, *in vivo*, and *ex vivo* studies (reviewed in McCarthy and colleagues,^(^ ^82^ ^)^ Mason and colleagues,^(^ ^83^ ^)^ and De Silva and colleagues.^(^ ^84^ ^)^) Compounds derived from vitamin D or the “overirradiation” product lumisterol have also been reported to reduce UV‐induced CPD in keratinocytes^(^ ^32^, ^33^, ^69^, ^70^ ^)^ and in mice.^(^ ^34^, ^38^ ^)^ The data presented here show for the first time that the CYP11A1 derivative of lumisterol, 24(OH)L_3_, reduces oxidative damage as well as CPD, provide a concentration response curve for 24(OH)L_3_ in comparison with 1,25(OH)_2_D_3_, and also show for the first time that this compound and 20(OH)D_3_, like 1,25(OH)_2_D_3_, reduces DNA damage in human keratinocytes when added immediately after UV exposure. Previous studies in collaboration with Tony Norman reported that both VDR and the endoplasmic reticulum protein ERp57 are required for reduction of UV‐induced CPD by1,25(OH)_2_D_3_,^(^ ^64^ ^)^ but the receptors through which 1,25(OH)_2_D_3_ reduces oxidative DNA damage and the receptors required by vitamin‐like compounds to reduce UV‐induced photo lesions were not known. The current study showed for the first time that both VDR and ERp57 are indeed required for reductions in oxidative DNA damage by 1,25(OH)_2_D_3_ and for the reduction in both types of UV‐induced DNA damage by the CYP11A1 metabolites, 20(OH)D_3_ and 24(OH)L_3_. The absolute requirement for VDR for the reduction in DNA damage, together with the apparent ability of these vitamin D–like compounds derived from vitamin D or lumisterol to reduce UV‐induced DNA damage, go some way toward explaining why knockdown of the VDR leads to increased susceptibility to photocarcinogenesis.^(^ ^50^ ^)^ In contrast, lack of the 1α‐hydroxylase does not apparently increase susceptibility to UV‐induced skin tumors.^(^ ^52^, ^85^ ^)^

Agents such as 1,25‐dihydroxylumisterol_3_, 20(OH)D_3_, and other vitamin D compounds made in skin,^(^ ^18^, ^19^, ^21^, ^38^, ^86^ ^)^ which have been shown to reduce UV‐induced CPD,^(^ ^32^, ^38^, ^41^, ^42^ ^)^ generally have reduced binding to the G‐pocket of the VDR in comparison to 1,25(OH)_2_D_3._^(^ ^58^, ^87^ ^)^ They interact with the VDR, however, and the addition of OH at C1α modifies such interaction.^(^ ^88^, ^89^, ^90^ ^)^ In this context, it is worth noting that some VDR, but not necessarily a fully functional VDR, seems to be required to enable 1,25(OH)_2_D_3_ to reduce CPD after UV, because this protective effect was shown with VDR which had a mutated ligand‐binding domain or a mutation in the DNA binding domain.^(^ ^64^ ^)^

In that study, we demonstrated the involvement of the endoplasmic reticulum stress protein, ERp57 (also known as PDIA3 or MARRS^(^ ^91^ ^)^) in the photoprotective response to 1,25(OH)_2_D_3._^(^ ^64^ ^)^ As well as siRNA to the protein, a neutralizing antibody to ERp57, which would not enter the cell, abolished the response, supporting the idea of a membrane position for this protein for this function.^(^ ^92^ ^)^ It is likely that the VDR was also present in the cell membrane as shown by the Norman group.^(^ ^57^ ^)^ Co‐immunoprecipitation studies of non‐nuclear fractions of human primary fibroblasts showed that ERp57 co‐immunoprecipitated with VDR and VDR with ERp57.^(^ ^64^ ^)^

The global importance of ERp57 was demonstrated in ERp57^−/−^ embryos where ERp57 deficiency was lethal at embryonic day 13.5, whereas embryos at embryonic day 12.5 were reported to be much smaller than the WT embryos.^(^ ^93^ ^)^ In the current study, in the presence of siERp57, which significantly reduced ERp57 expression, the reduction of UV‐induced CPD and 8‐OHdG by 1,25(OH)_2_D_3_, 20(OH)D_3_, and 24(OH)L_3_ was also significantly reduced. Further studies of the role of ERp57 in photoprotection will require the use of animals that have a conditional knockout of this protein in the epidermis.

The current study also showed that when keratinocytes had been incubated with tunicamycin, a known inhibitor of glycosylation,^(^ ^77^ ^)^ the protection by 1,25(OH)_2_D_3_ against UV‐induced CPD was abolished and that against 8‐OHdG was impaired. The explanation for this finding remains unclear and warrants further investigation. In that set of experiments, the tunicamycin was actually used to inhibit glycosylation of the calcium‐sensing receptor in order to prevent its movement to the cell membrane. The 1,25(OH)_2_D_3_ was included as a positive control for DNA damage reduction. Glycosylation is not apparently a feature of posttranslational modification of VDR, though slightly higher molecular weight bands have been reported in some tissues,^(^ ^94^ ^)^ which could be a result of posttranslational modification.^(^ ^95^ ^)^ These authors reported hyperglycemia‐induced enzyme mediated glycosylation (O‐linked‐N‐acetylglucosaminylation, or OGlcNAcylation) of VDR.^(^ ^95^ ^)^ Tunicamycin competes with N‐acetylglucosamine phosphotransferase to inhibit N‐glycosylation^(^ ^77^ ^)^ and would not be expected to inhibit OGlcNAcylation. A search of the literature revealed no evidence of glycosylation of ERp57. Nevertheless, it is possible that tunicamycin interferes with some chaperone protein important for translocating either VDR or ERp57 to the plasma membrane. Testing this proposal was beyond the scope of the current study, but the observations support, at least in part, the hypothesis that membrane associated VDR and/or ERp57 is important for some aspects of photoprotection. Tunicamycin also triggers endoplasmic reticulum stress^(^ ^96^ ^)^ and is thus is likely have an effect on a typical stress marker, such as ERp57.^(^ ^97^ ^)^

As noted in the second paragraph of the discussion, 1,25(OH)_2_D_3_ reduced UV‐induced CPD in fibroblasts with DNA binding domain mutations of the VDR.^(^ ^64^ ^)^ 1α,25(OH)_2_‐lumisterol_3_ has little ability to modulate gene transcription, but was shown to initiate nongenomic action with a similar potency to 1,25(OH)_2_D_3._^(^ ^58^, ^98^ ^)^ 1α,25(OH)_2_‐lumisterol_3_, similar to 1,25(OH)_2_D_3_, reduced UV‐induced CPD in human primary fibroblasts and keratinocytes^(^ ^29^, ^32^ ^)^ and reduced photocarcinogenesis in Skh:hr1 mice, though not to the extent seen with 1,25(OH)_2_D_3._^(^ ^34^ ^)^ All these data and the involvement of ERp57 support the proposal that photoprotection by 1,25(OH)_2_D_3_ and other vitamin D–like compounds at concentrations similar to 1,25(OH)_2_D_3_ signal, at least in part, by nongenomic pathways. The chloride channel inhibitor, DIDS, inhibited UV‐induced CPD reduction by 1,25(OH)_2_D_3_ and 20(OH)D_3_, further supporting involvement of a nonclassical vitamin D pathway in the reduction of UV‐induced DNA lesions by these vitamin‐D like compounds.^(^ ^37^, ^99^ ^)^

Some of the actions of 1,25(OH)_2_D_3_ shown here that could contribute to protection from UV damage and subsequent photocarcinogenesis seem unlikely to be mediated by changes in gene transcription. These include suppression by 1,25(OH)_2_D_3_ of otherwise upregulated phosphorylation of protein kinase B (AKT) (acutely transforming retrovirus AKT8 in rodent T‐cell lymphoma) and extracellular regulated kinase‐1/2 (ERK1/2), after UV.^(^ ^36^ ^)^ 1,25(OH)_2_D_3_ also increased phosphatase and tensin homolog (PTEN) expression after UV.^(^ ^100^ ^)^ UVB normally downregulates PTEN in cells and whole skin in an AKT‐dependent and ERK1/2‐dependent manner.^(^ ^101^ ^)^ In turn, PTEN downregulation impairs global genomic nucleotide excision repair, which is needed to remove UV‐induced DNA lesions such as CPDs.^(^ ^102^ ^)^ The 1,25(OH)_2_D_3_–induced reduction in ROS as soon as 15 minutes after UV probably contributes to reduced oxidative DNA lesions and is likely to result in less damage to repair enzymes and thus to enhance repair.^(^ ^36^ ^)^ Likewise, after UV, the 1,25(OH)_2_D_3_–induced reduction in RNS and damaging nitrosylation of proteins, including DNA repair enzymes, is likely to facilitate DNA repair.^(^ ^37^ ^)^

In the current study, we observed a reduction in oxygen consumption rate, indicative of a reduction in oxidative phosphorylation, in both sham and UV‐exposed keratinocytes. We had previously reported that energy from glycolysis was required for DNA repair,^(^ ^36^ ^)^ and it is clear from the results presented here that even treatment with a well‐known inhibitor of oxidative phosphorylation, oligomycin, had no effect of the ability of 1,25(OH)_2_D_3_ to reduce DNA lesions. On the other hand, increased activity of the electron transport chain in later stages of oxidative phosphorylation has been shown to be critically important for induction of skin cancers in mice by UV.^(^ ^80^ ^)^ If this process is blocked, actinic tumors do not develop.^(^ ^80^ ^)^ Furthermore, agents that interrupt the mitochondrial respiratory chain, such as metformin,^(^ ^80^ ^)^ and it seems from the current data, 1,25(OH)_2_D_3_, reduce tumorigenesis.^(^ ^34^, ^103^ ^)^

As observed in this study, increased phosphorylation of CREB at Serine^133^ occurs after UV exposure. This increased CREB phosphorylation seems to be important in the early stages of skin tumor development in mice.^(^ ^81^ ^)^ There is some evidence that the increased CREB phosphorylation after UV is at least, in part, downstream of increased ERK1/2 phosphorylation.^(^ ^104^ ^)^ So the reduction in ERK1/2 phosphorylation after UV with1,25(OH)_2_D_3_, reported earlier,^(^ ^36^ ^)^ may partly explain the reduced CREB‐phosphorylation after UV noted here. In turn, reduced phosphorylation of CREB‐Ser^133^ even when the reduction is relatively modest as seen here with 1,25(OH)_2_D_3_ and as described for the plant‐derived flavonol, kaempferol, are enough to significantly suppress UV‐induced skin tumor development.^(^ ^34^, ^105^ ^)^

## Conclusion

Although Professor Anthony W. Norman did not live to see the extension of his work in photoprotection, the accumulating data further supports the ideas he promulgated for mechanisms of action of the vitamin D hormone, including a role for membrane VDR and nonclassical effects on various cell signaling pathways, as well as the classical steroid hormone modulation of gene transcription.

## Conflict of Interest

None.
